# Practical Notes and Formulæ

**Published:** 1888-01

**Authors:** 


### PRACTICAL NOTES AND FORMULÆ.

**Insomania:**

℞. Potassii bromidi..................................

Hydrat. chloral..............................aa ʒ iij

Morphiæ sulph................................gr. j

Syr. tolutani.......................................

Aq. menth. pip..............................aa ℥ ij

One teaspoonful every hour until asleep.—*Medical World.*

**Congestive Chill:**

℞. Tinct. opii.................................♏ xx

Chloroformi...................................♏ xx

In water in violent congestive chill. Or

℞. Morphiæ.......................................gr. 1-6

Atropiæ..........................................gr. 1-40

Subcutaneously.—*Ib.*

**Congestive Chill:**

℞. Iodine..............................................ʒ j

Potass. iodidi.......................................ʒ ij

Aq. dest..............................................℥ jss

Five drops in water every two hours in chills.—*Medical World.*

**Dysentery.**—Dr. Jones (in *Medical World*) writes: If Dr. Hudson, of Bethel, Tenn., will try the following prescription for dysentery, he will have but little trouble in controlling it, providing he sees the patient within the first few days after the attack:

℞. Sulph. magnes...................................℥ j ss

Calcined magnes...................................ʒ ij

Ginger...................................................ʒ j

Ipecac...................................................gr. x

Mix. Dose.—A teaspoonful every hour and a half until it produces free watery discharges from the bowels, then withold treatment. Should a relapse to small dysenteric discharges occur, repeat as before.

After the condition of the bowels is changed, I frequently follow with the following as a tonic:

℞. Quinine...............................................gr. xx
Leptandrin.............................................gr. x
Hydrastis................................................gr. v
Subnitrate of bismuth.................................gr. xx

Mix, and divide into ten powders. Dose.—One powder once in three or four hours.

The above prescriptions are for an adult. Change dose according to age.

I have been using this treatment for dysentery twelve years, with perfect success.

**Good Samaritan Liniment.**——Good Samaritan Liniment is made as follows:

℞. Oil of sassafras.......................f ℥ 2
Oil of wintergreen.......................f ℥ 2
Oil of thyme.......................f ℥ 2
Oil of ambe.......................f ℥ ii
Oil of cedar.......................f ℥ ii
Oil of origanum.......................f ℥ xii
Oil of peppermint.......................f ℥ ii
Oil of hemlock.......................f ℥ iv
Balsam of fir.......................f ℥ ii
Sulphuric ether.......................f ℥ ii
Alcohol.......................gall. v

Mix.

**Remedy for Chronic Malaria** :

In all cases characterized by enlarged liver and spleen, or in which neuroses are prominent the following treatment has usually been efficacious :

℞. Tinct. iodinii comp.......................ʒ iss

Sol. Fowleri.......................*m.* xlviii

Syrupi sarsaparilla comp.......................q. s ad f ℥ iii

S. Teaspoonful three times a day after meals in one-half tumbler of water, at bed time.

Also an ointment like the following :

℞. Ungt. hydrag. nitratis.......................℥ i
Ext. belladonnæ.......................ʒ i

M. S. To be rubbed over liver and spleen once daily.—*Medical and Surgical Synopsis.*

**For Chafing Infants** :

M. Lorenz, in his "*Ichthyol in de: Chirurgic*" recommends the following, which he declares acts promptly :

℞. Ammon. Sulph. Ichtholiici.......................gr. iii
Unguent paraffine.......................ʒ ii
Cumarine.......................gr. vii–xv

M. Apply to the chaps, first bathing the child.—*St Louis Medical and Surgical Journal.*

**A neat** and convenient way to handle *corrosive sublimate* for making antiseptic solution is to dissolve 15 grains in fʒj of alcohol, which, added to a quart of water, makes 1-1000, and does not undergo chemical change, if used immediately.—*College and Clinical Record.*

**Aperient Pill** :

The following aperient pill has been used by Professor Louis Bauer for the last thirty-five years, with unvarying satisfaction :

℞. Ext. Hyoscyami .......................... gr. x
  " Aloes aquosi ..............................
  " Colocynth comp. (Squibb) ................ ℈ i
 Sod. et pot. tart. ............................ ʒ ss

M. Divide in 20 pills, more or less, to be taken at a time.

The object being to secure a physiological action of the bowels, and no purging. The pills should be prepared in small quantities, being more effectual than when kept for a length of time.

When the pill is put in capsules their effect is longer maintained. Squibb's extract of colocynth is indispensable to their reliability, because it contains scammony and not gamboge. The latter is worthless, causes griping and not the action intended.—*Medical and Surgical Journal.*

**Treatment of Acne.**—Lassar recommends, in the *Therapuetische Monatshefte*, 1887, No. 1, the application of Wilson's ointment, prepared as follows :

℞· B-naphthol ............................... parts x

 Sulphur præcipitat ......................... parts l

 Vaseline (or lanolin) ........................

 Saponis virid ............................ aa parts xxv

M. Rub gently together to make a paste.

This ointment is to be smeared on in a layer as thick as the back of a table-knife, and carefully removed, after thirty minutes, with a soft rag. After this the part is to be powdered with talc.—*Centralblatt fur Chirurqie*, Oct. 1, 1887.

For a case of *gastro-intestinal catarrh*, Professor DaCosta ordered broth diet and a presciption containing :

℞. Bismuth. subnit ............................ gr. x
 Pulv. aromatic .............................. gr. iij
 Pulv. opii .................................... gr. ¼
 Ft. chart j ....................................
M.

Sig. Take four times a day.—*College and Clinical Record.*

The following prescription has heen used with favorable results in general *constipation* among the patients of the out-door department of the Jefferson Hospital :

℞. Ext. cascaræ fluid ..........................
 Ext. glycyrrhizæ fluid ..................... aa f℥j M

Sig. Teaspoonful at bedtime.—*College and Clinical Record.*

